# COVID-19 vaccines: history of the pandemic’s great scientific success and flawed policy implementation

**DOI:** 10.1007/s40592-024-00189-z

**Published:** 2024-03-09

**Authors:** Vinay Prasad, Alyson Haslam

**Affiliations:** Department of Epidemiology and Biostatistics, University of California San Francisco, 550 16 St, 2 Fl, San Francisco, CA 94158 USA

**Keywords:** COVID-19, Health policy, Vaccinations

## Abstract

The COVID-19 vaccine has been a miraculous, life-saving advance, offering staggering efficacy in adults, and was developed with astonishing speed. The time from sequencing the virus to authorizing the first COVID-19 vaccine was so brisk even the optimists appear close-minded. Yet, simultaneously, United States’ COVID-19 vaccination roll-out and related policies have contained missed opportunities, errors, run counter to evidence-based medicine, and revealed limitations in the judgment of public policymakers. Misplaced utilization, contradictory messaging, and poor deployment in those who would benefit most—the elderly and high-risk—alongside unrealistic messaging, exaggeration, and coercion in those who benefit least—young, healthy Americans—is at the heart. It is important to consider the history of COVID-19 vaccines to identify where we succeeded and where we failed, and the effects that these errors may have more broadly on vaccination hesitancy and routine childhood immunization programs in the decades to come.

## Introduction

The COVID-19 vaccine has been a miraculous, life-saving advance, offering staggering efficacy in adults, and was developed with astonishing speed. The time from sequencing the virus to authorizing the first COVID-19 vaccine was so brisk even the optimists appear close-minded. Yet, simultaneously, United States’ COVID-19 vaccination roll-out and related policies have contained missed opportunities, errors, run counter to evidence-based medicine, and revealed limitations in the judgment of public policymakers. How can a single intervention simultaneously represent one of our greatest pandemic successes but also encapsulate real limitations? Misplaced utilization, contradictory messaging, and poor deployment in those who would benefit most—the elderly and high-risk—alongside unrealistic messaging, exaggeration, and coercion in those who benefit least—young, healthy Americans—is at the heart. It is important to consider the history of COVID-19 vaccines to identify where we succeeded and where we failed, and the effects that these errors may have more broadly on vaccination hesitancy and routine childhood immunization programs in the decades to come.

## Breakthrough results in adult volunteers

On Nov 9, 2020, Pfizer press released results (Pfizer 2020a) of their ongoing, adult COVID-19 randomized control trial. This trial randomized over 40,000 individuals to the Pfizer-BioNTech vaccine or placebo and showed, after 94 infections, a large, 90% plus reduction in symptomatic COVID-19, among volunteers who did not have COVID-19 at baseline.

A week later, Moderna reported similar results (*Moderna’s COVID‑19 Vaccine Candidate Meets its Primary Efficacy Endpoint in the First Interim Analysis of the Phase 3 COVE Study | Business* *Wire *2020). *Moderna’s COVID‑19 Vaccine Candidate Meets its Primary Efficacy Endpoint in the First Interim Analysis of the Phase 3 COVE Study | Business Wire* (2020) Their ongoing Phase 3 COVE trial randomized over 30,000 participants to vaccine or placebo. With 95 cases of symptomatic covid, the cases split: 90 in the placebo arm and 5 in the vaccine arm, yielding a 94% reduction in symptomatic COVID-19. The Moderna trial further bolstered claims of efficacy by showing that, among 11 cases of severe disease, all occurred in the control arm. By the time the trial was ultimately published, 30 severe covid cases would occur, including 1 death – all in the control arm.(Baden et al. 2021).

It was clear, by the fall of 2020, COVID-19 vaccines could reduce symptomatic COVID-19 and also severe disease against prevailing strain(s) in adults. This finding will be remembered as a seminal moment in medical history, and two of these products rapidly received emergency use authorization (EUA) in the US. Pfizer received EUA on Dec 11, 2020 for ages 16 and up (Pfizer 2020b). Moderna received EUA on Dec 18, 2020 for 18 and up (Mezher 2020) with distribution following soon thereafter.

## Sowing doubt in the months prior to emergency use authorization

The months preceding the press-releases were anything but optimistic. Top US outlets as well as medical and scientific journal articles prior to Nov 2020 continually undermined COVID-19 vaccine prospects, cast doubt on the FDA’s regulatory standards, and articulated talking points that, to this day, remain co-opted by anti-vaccine groups.

On June 8 2020, two senior physicians from the University of Pennsylvania wrote an op-ed warning of Trump’s potential October surprise—the idea the president would debut a vaccine that did not meet high standards for safety or efficacy prior to the election, in order to boost his political odds (Emanuel and Offit 2020). The authors drew a comparison to previously approved vaccines, RotaTeq and Rotarix vaccines (for rotavirus), which enrolled 70,000 and 63,000 children, respectively, and took 4 + years to establish safety, efficacy, and obtain approval. The COVID-19 vaccine studies, in contrast, were slated to enroll fewer participants and to be completed in far less time.

The authors wrote, “even if a vaccine generates antibodies, it does not prove that the vaccine is effective at preventing infection; it only makes it more likely that the vaccine would be effective.” The article cautioned that with just 20,000 participants receiving the vaccine, “serious but rare side effects might be missed.”

On August 5, 2020, an essay appeared on the British Medical Journal (BMJ) opinion website entitled, “The rush to create a COVID-19 vaccine may do more harm than good” (Torreele 2020). The article lamented the fact that a vaccine may only provide short term protection or may offer only low vaccine effectiveness (less than 50%). The article quotes Phil Krause, then deputy director of vaccines at the US FDA, who said, “A weakly effective vaccine can do more harm than good.” The BMJ piece also quotes Ken Frasier, CEO of Merck, who said those “raising hopes for a vaccine before year-end are doing ‘a grave disservice to the public.’”

The idea that we were “rushing a vaccine” was common in media coverage throughout 2020. On Sept 4, 2020, Donald Trump promised to, “produce a vaccine before the end of the year, or maybe even sooner” (Reich and Masket 2020). Trump added, “Nobody thought it could be done this fast. Normally it would be years, and we did it in a matter of a few months. We are producing them in advance so hundreds of millions of doses can be quickly available. We have a safe and effective vaccine this year, and together we will crush the virus.”

Yet, reporters cautioned skepticism. A Washington Post article described the 1976 swine flu vaccine debacle that led to hundreds of cases of Guillain- Barré, a paralytic condition, and a product that was ultimately withdrawn. The article noted the FDA’s recent embrace of EUA (for hydroxychloroquine and convalescent plasma—two therapies supported by weak or absent data) and how this could further undermine vaccine confidence.

Another piece (Sept 10, 2020), echoed these concerns. Writing for CNN, an expert New York City physician lamented that, “the history of vaccines is full of alarming missteps” (Sepkowitz 2020). The piece detailed examples of the erroneous administration of the wrong tuberculosis vaccine, and an unapproved polio vaccine. It revisited the 1976 swine flu vaccine debacle and detailed a 1998 blunder with an ultimately failed Lyme disease vaccine. On Oct 16, 2020, Carl Zimmer, writing for the New York Times, wrote “some vaccines may be abruptly withdrawn from the market because they turn out not to be safe” (Zimmer 2020).

Some scientists argued that the FDA had asked companies to focus on incorrect outcomes. Writing with a colleague, cardiologist Dr. Eric Topol, penned a New York Times op-ed on Sept 22, 2020 suggesting COVID-19 vaccines should show evidence of lowering severe illness (Doshi and Topol 2020). Symptomatic disease, including milder forms of illness, was the primary endpoint of ongoing trials.

A Washington post op-ed, by William Haseltine, entitled “Beware of covid-19 vaccine trials designed to succeed from the start” voiced similar concerns (Haseltine 2022). Dr. Haseltine advanced the idea that the vaccines could have unknown side effects that occur years after approval. Haseltine wrote, “Rushed Moderna and Pfizer trials could bring about similar short-term health consequences or, potentially far worse, lead to long-term health consequences that we won’t discover until months or years after the vaccine’s approval.”

**Fig. 1:**
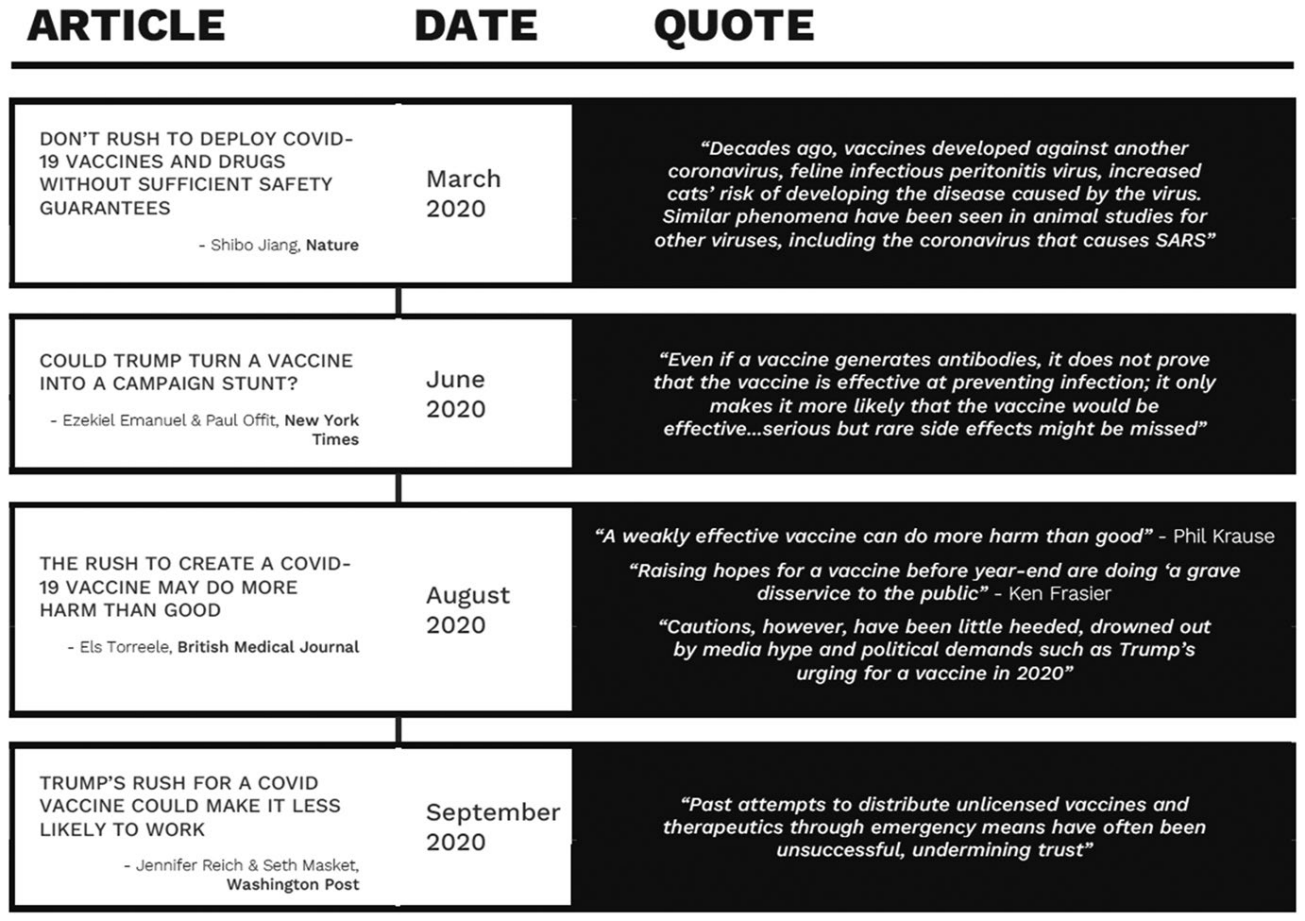
Timeline of quotes regarding vaccine roll-out

The idea that vaccines could have negative consequences was articulated early in the pandemic. In March of 2020, in the journal *Nature*, Shibo Jiang, a vaccine researcher wrote an article entitled, “Don’t rush to deploy COVID-19 vaccines and drugs without sufficient safety guarantees” (Jiang 2020). In it, he noted that it was possible for a COVID-19 vaccine to make it easier to get COVID. Jiang explains, “Decades ago, vaccines developed against another coronavirus, feline infectious peritonitis virus, increased cats’ risk of developing the disease caused by the virus. Similar phenomena have been seen in animal studies for other viruses, including the coronavirus that causes SARS.” A detailed timeline of these quotes appears in Fig. 1.

An article in the Lancet from October 27, 2020, echoed this concern regarding adenoviral vector vaccines (AstraZeneca and Johnson & Johnson) (Buchbinder et al. 2020).

On Sept 23, 2020, the National Vaccine Advisory Committee advised against the use of EUA (a lower regulatory hurdle), and instead urged the use of Biological Licensing Agreement to clear a COVID-19 vaccine, a process that would delay approval many months (Jenco 2020).

Peter Hotez, founding dean of the National School of Tropical Medicine at Baylor College of Medicine and a later co-developer of SARS/MERS/SARS-2 vaccines, wrote a Twitter thread in Sept 2020 citing a “dozen reasons” why he was skeptical of using EUA – the regulatory path used initially by all covid 19 vaccines (Hotez@PeterHotez 2020). He specifically called the pathway, “a substandard or lesser review” process than the more traditional biological licensing agreement.

Ashish Jha, at the time a professor of medicine and frequent television pundit, who would later become the Biden COVID czar and lead the US COVID Task Force (April 15, 2022) (Florko 2022) said that commissioner Steve Hahn was “suggesting he could issue EUA for a vaccine if benefit > risk.” Jha writes, “That is totally inappropriate Unlike therapies, which are given to sick people, vaccines are given to healthy people Needs a higher bar Full (expedited) review after completed phase 3” (Jha@ashishkjha 2020).

Public perception of vaccines deteriorated over the summer of 2020. By mid-September 2020, Pew research showed trust in vaccines had dropped since polling from May of that year (Tyson et al. 2020). As of September, 78% of respondents felt that the greater risk was moving too fast with vaccination, rather than too slow, and 77% felt a vaccine would be approved before its safety and efficacy was fully understood. Both Republicans and Democrats were less likely to get the vaccine than in prior polls, with Republicans displaying greater reluctance than Democrats. A Gallup poll from September (eventually published in the Journal of the American Medical Association) confirmed that Republicans were less likely to seek vaccination and more likely to oppose mandates (Largent et al. 2020).

The months preceding the successful press releases from Pfizer and Moderna witnessed juxtaposed messaging: ranging from sensational, optimistic, and promising statements by President Trump, to a concern that vaccines may be approved without sufficient safety and efficacy data from the media, politicians, and scientists. Key talking points included: stories of prior failed vaccine products, claims that long-term safety would not be known at product launch, suggestions that a COVID vaccine could theoretically enhance illness or viral acquisition, claims that 20,0000 participants were insufficient to exclude rare safety signals, and arguing that the EUA process was substandard, and perhaps should not be utilized. Ironically, several of these points would prove salient to subsequent vaccine and booster authorizations, particularly in young populations, and the emerging understanding of vaccine induced myocarditis, while other points would be co-opted or misused to justify vaccine hesitancy among older adults in the months that followed.

It is difficult to separate what portion of skepticism was scientifically motivated—due to the testing of a hitherto novel vaccination platform (the mRNA technology) on an unprecedented time-scale (mere months) with implications for the fate of world and global economy—and how much was politically motivated—distrust of a divisive sitting US president, Donald J Trump, who faced a contentious re-election campaign, who was repeatedly one of the vaccines’ most passionate proponents, and whose administration launched the Operation Warp Speed program, which was, in part, responsible for the rapid development.

Regardless, trust in the forthcoming vaccines was shaped by the conflicting and shifting messaging, and some of these players moved from a commentator role to more official advising. Confidence may have been strengthened if there had been a clear regulatory pathway set, which outlined outcomes and benchmarks for approval during a pandemic. Moreover, a more balanced discussion of the risks and benefits of the vaccine may have been achieved with input from a breadth of experts.

## The FDA and companies delay vaccine trial until after the US election

One major point of contention throughout 2020 was when vaccine trial results would be reported and when a vaccine would be distributed. Trump repeatedly felt that this would occur as early as October 2020 and certainly before the end of the year (NPR 2020), while ultimately the results were released on Nov 8, 2020, and public vaccination began in December.

An *MIT Technology Review* article notes that some physicians have taken credit for delaying the trial results until November. The piece was entitled “One doctor’s campaign to stop a covid-19 vaccine being rushed through before Election Day: How heart doctor Eric Topol used his social-media account to kill off Trump’s October surprise” (Regalado 2020). The piece describes several tactics used to delay approval.

Topol and 60 experts sent an open letter to Albert Bourla, CEO of Pfizer, asking for a minimum of 2 months of follow up for each enrolled volunteer in vaccine trials, a change of protocol that would ensure approval could not occur until after the November election (*Letter to Pfizer* 2020). The letter did not explain why 2 months per participant would enhance safety—in contrast with a median of 2 months follow-up, meaning some participants could be followed for less time, as long as others were followed for more time. After the letter was publicized, according to Politico, Albert Bourla and Eric Topol met to discuss concerns of approval prior to the election (Cancryn and Owermohle 2020).

The FDA did change its guidelines for EUA of COVID-19 vaccines in September of 2020, (Zimmer and Weiland 2020) but ultimately decided upon 2 months of median follow-up (not the Topol letter’s suggestion). It also required at least 5 cases of severe disease in the control arm. This requirement had potential to extend trial duration.

Clinical trials may stop after a pre-defined number of events, in this case, symptomatic SARS-CoV-2 infections, and after statisticians have examined the rates by arm. If events are extremely skewed, i.e., exclusively happening in 1 arm or the other, a trial can be deemed statistically persuasive and be halted, even if the raw number of events are few. On Sept 9, 2020, Eric Topol interviewed Dr. Paul Offit for the website Medscape (Topol and Offit 2020). During the interview (per transcript), Dr Topol seemed shocked a trial could be persuasive with 100 events. He said:

“But this week, for example, the Pfizer CEO said they could demonstrate efficacy with very small numbers (Herper 2020a) of cases in the placebo and vaccine groups; these numbers seem totally out of line with what would be considered stopping rules. I mean, you're talking about giving a vaccine with any of these programs to tens of millions of people. And you're going to base that on 100 events?”

Dr. Offit explains that it is not the raw event numbers, but the distribution across arms that matter; one could have a significant trial even with few events, “assuming that you have just a handful or fewer people in your vaccine group who were ill.” And again, Offit replies, “I think one could only imagine stopping with 160 cases if you have virtually no cases in your vaccine group.”

Ultimately, when Pfizer announced results on Nov 8th, the company noted it had “recently elected to drop the 32-case interim analysis and conduct the first interim analysis at a minimum of 62 cases” (Pfizer 2020a). By the time the company analyzed results, 94 cases had occurred.

An article in the journal *Science* tried to dismiss the allegation that the number of events was altered merely to delay the vaccine trial result until 5 days after the ongoing US presidential election (Cohen 2020). The article contends that COVID19 events were accruing rapidly, and given that 62 events would occur shortly after, this was preferred for improved “statistical power”. But this explanation is problematic because 32 events was carefully chosen, and the distribution of events was deemed a priori to be mathematically persuasive if it were reached. Specifically, the trial would only be halted if fewer than 6 events of 32 (6 vs 26) occurred in the vaccine group. With fewer total events (Herper 2020b), the skew would have to be more extreme to halt a trial than with far more events to preserve the statistical confidence.

Second, adherence to a pre-planned statistical plan is far better for confidence than any protocol deviations or modifications—for this precise reason: doubt can exist about the true motives. The same *Science* article discusses how because of the ongoing changes, Pfizer had stopped testing swabs to avoid a protocol deviation, which resulted in 94 events occurring before testing and a further delay in results. And third, FDA officials postulated that between announcing trial results and FDA meeting further events would occur, likely raising the total number of events to over 100 (Herper 2020b).

These changes (minimum severe disease case requirement and change in interim analysis) delayed the eventual trial results. It is unclear if these design features enhanced the scientific validity of the trial. It is also unclear on what specific day results would have been reported had it not been for changes.

## The vaccine is rolled out, poorly

By December 2020 and into January 2021, vaccination began. Three decisions illustrate suboptimal vaccine deployment.

First, the US over prioritized young essential workers, amidst a constrained vaccine supply (Stolberg and LaFraniere 2021). While the UK prioritized nursing home residents and older individuals (> 80) (Warren and Pogkas 2020), the US included essential workers, including young, resident physicians. Of course, health care workers face higher risks of acquiring the virus due to occupation (though this was and is offset by available personal protective equipment), but this was less than the elevated risk of death faced by older individuals. In other words, while the increased risk of occupational exposure was on a linear scale, the increased risk of poor outcomes by age grew exponentially.

This prioritization scheme was developed with two opposing ethical considerations—one, to reduce infection among workers who were more likely to be poor and people of color and who had also been disproportionately affected by death and infection, and two, to reduce death among those with serious medical conditions and were most likely to die (Goodnough and Hoffman 2020). In retrospect, the prioritization of essential workers was insufficient to reduce racial/ethnic disparities in vaccine uptake (Nicholas et al. 2022).

Second, actions taken by the USA repeatedly pushed to vaccinate young people prior to vaccinating older people globally (Høeg et al. 2021). This was in part because of the lower than expected uptake among older adults, but the advice was based on infections and did not consider differences in serious outcomes, where younger people are much less likely to have serious outcomes when infected. Yet, this approach was in contradiction to that of the World Health Organization (WHO) (Miller 2021). The WHO specifically stated on Nov 21, 2022: (World Health Organization 2021).

“As a matter of global equity, as long as many parts of the world are facing extreme vaccine shortages, countries that have achieved high vaccine coverage in their high-risk populations should prioritize global sharing of COVID-19 vaccines through the COVAX facility before proceeding to vaccination of children and adolescents who are at low risk for severe disease.”

Third, the US insisted that a 1 dose first vaccination strategy (Wachter and Jha 2021)—where dose 2 would be delayed, in order to give more individuals a first dose in the setting of a constrained supply—should not be pursued. This decision would ultimately prove erroneous. A number of studies (Romero‑Brufau et al. 2021) ultimately established that delaying (Tuite et al. 2021) the second dose would have lowered population case rates and COVID-19 mortality, and increased antibody production (Martinez and Ooi 2022) and possibly durability. The United Kingdom did move forward with a 1 dose strategy, a decision that analysts believe has been totally vindicated (Lovett 2021).

Lessons from these decisions could inform future policy. Future a priori rollout plans should, of course, consider disease-specific risks and benefits in all population subgroups, but also consider outcomes that are most important for the population (e.g., death or infection) and whether there can be effective modifications to how the vaccine is administered.

## Johnson and Johnson Vaccine Leads to Vaccine Induced Thrombocytopenia and Thrombosis

The third entrant to the US market was the Johnson and Johnson (J&J) adenoviral vector vaccine. The product was notable for requiring only a single dose, and was authorized on Feb 20, 2021 (Johnson and Johnson 2021). The product was supported by the phase 3 ENSEMBLE study which found a 67% reduction in the primary endpoint of symptomatic SARS-CoV-2 (Sadoff et al. 2021). As the third entrant into the market, J&J never enjoyed the market share of its predecessors, but due to easier temperature requirements and a single dose administration, it offered some advantages. Yet, very quickly after product launch, an important safety signal would emerge.

In mid-April 2021 (Prasad 2021a), a series of unprecedented vascular events were noted, mostly in women, shortly after receipt of the J&J vaccine, which prompted the FDA to issue a pause while it investigated. In the weeks that followed, it became clear that a real and unanticipated side effect of the adenoviral vector vaccines was vaccine induced thrombocytopenia and thrombosis (VITT)—a condition with runaway platelet activation and profound clotting. After being given the J&J vaccine, several young women died or were neurologically devastated (Greinacher et al. 2021).

On social media, efforts were made to downplay the concern, and memes emerged contrasting the risk of clotting after J&J vaccines to clots after oral contraceptives (Prasad 2021a; Gray 2021; Rasmussen@angie_rasmussen 2021; Prasad 2021a). However, these were in no way analogous, as the vaccine’s clots were noted to occur in the cerebral veins, and in the setting of runaway platelet activation—a far more dangerous hematologic condition than a blood clot in a lower limb. Some sought to subtract baseline rates of cerebral vein clots from population estimates of vaccine-induced prothrombotic immune thrombocytopenia (VITT). This was inappropriate because VITT is an entirely novel hematologic ailment, of which there is no baseline rate. Cerebral venous thrombosis in isolation is distinct from the same clot in the setting of runaway platelet activation that denotes VITT.

On April 15th, 2021, (@VPrasadMDMPH (Vinay Prasad) 2021) I argued that, given the presence of alternatives (Pfizer and Moderna), that it was “game-over” for J&J in women less than 50 years old. The US should halt its use. Yet, the FDA released the pause and took no major action until May 2022 (a full year later) (Branswell 2022), when it changed the label for J&J restricting it only to those who cannot take another vaccine for medical reasons. This 1-year delay led to unnecessary vaccine-induced injury.

Two lessons from the J&J fiasco carry implications going forward. First, vaccine regulation can be quick to authorize in times of crisis, but far slower to address serious safety concerns. This remains a problem. And second, in a new year, and with a new president, media and pundits would repeatedly err on the side of downplaying vaccine concerns, while before they had exaggerated them.

## Myocarditis from mRNA vaccines in young, healthy men

The first reports that myocarditis (Prasad et al. 2021)—an inflammation of the heart muscle—may be an important safety concern for mRNA vaccination emerged in the Jerusalem Post in February 2021 (Jaffe‑Hoffman 2021). By April 25, 2021, Reuters had picked up the story (Reuters 2021a). Yet, on April 27, 2022, the CDC commissioner specifically denied having found a link (Reuters 2021b), "We have not seen a signal and we've actually looked intentionally for the signal in the over 200 million doses we've given." The European Medicines Agency launched an inquiry on May 7, 2021 (European Medicines Agency 2021). By May 22, 2021, the CDC had reversed course and announced it had received reports and encouraged providers to send in more (Mandavilli 2021).

As of 2022, accumulating evidence has found that mRNA vaccines are associated with myo- and pericarditis (Buchan et al. 2022). This occurs most often in young men (aged 12–30), with peak incidence in those 16–24 years of age. It occurs more often with dose 2 than dose 1, and some studies suggest it occurs more often with the Moderna product than Pfizer’s. It also occurs after boosters (3rd doses) (Sharff et al. 2022). As such, several European nations have restricted Moderna in young people (Reuters 2021d), as early as October 2021 (Taylor 2021).

Myocarditis changed the COVID-19 vaccination calculus, yet the US never adequately responded. The goal of vaccination programs is to maximize the benefit of vaccination and minimize the harm. Now, a clear safety signal has emerged in a target demographic. This demographic faces far lower risks from SARS-CoV-2 than older ages and yet now faces non-trivial safety concerns. In June of 2021, my colleagues and I argued that the CDC’s “all or nothing” approach to vaccination may be misguided (Prasad et al. 2021).

Our analysis was simple. Some estimates suggest the first dose alone provides 85–95% reductions in hospitalization (Andrews et al. 2022). In that case, do young men truly benefit from dose 2? Or do the harms outweigh the benefits? One analysis suggests maybe not, at least for healthy adolescents (Krug et al. 2022). Or, alternatively, what if the doses were spaced further apart? What if lower doses were tried in young men? Current schedules use 100 µg × 2 (Moderna), or 30 µg × 2 (Pfizer) in both 20-year-old men and 80-year-old women. Is this optimal? Should vaccination guidelines vary between young people with comorbidities and those who are completely healthy? And most importantly, should the guidelines be altered for young people who have had and recovered from COVID-19? In other words, do infections count as 1 or 2 doses?

The way to answer these questions with highest scientific accuracy is to demand Pfizer and Moderna conduct a randomized controlled trial for each question. There are hundreds of thousands of individuals who struggle with each of these dilemmas. Moreover, both Moderna and Pfizer have profited tremendously from the pandemic and have the resources to resolve these uncertainties. Yet, neither the CDC nor FDA have demanded companies complete these trials. The FDA did demand Pfizer collect random cardiac enzymes after vaccination to identify the extent of subclinical myocarditis (Approval letter: BNT162b2 2021), but the company has yet to satisfy this requirement.

In the absence of demanding and rigorous trials, the CDC is not impotent. The agency could experiment in uncontrolled fashion. Try any of these strategies and follow young men to see if their COVID-19 outcomes were favorable. As yet, a third alternative, the CDC could make changes to mitigate harm—such as limiting Moderna or spacing doses—based on precaution.

Notably, Norway initially spaced doses in adolescents at 12 weeks, and only later permitted doses to be given 9 weeks apart (Reuters 2021c). Later the agency said adolescents could get 1 dose if they wished (Government.no 2022), adding, “the greatest benefit has already been achieved by taking the first dose, and a second dose entails a slightly elevated risk of myocarditis.” This logic was later extended to their 5 to 11 year old guidance (Norwegian Institute of Public Health 2022). The US meanwhile waited until February 2022 to allow spacing the doses up to 8 weeks apart (Gumbrecht and Christensen 2022), but it has taken no other measure to lower the risk of this unfortunate adverse event. Rhetoric has repeatedly sought to discount the concern rather than ameliorate it.

As such, the US failed the social contract of accelerated vaccine authorization, thus eroding public trust. While emergency use authorization makes sense in dire and critical situations, there must be an equal effort to act expeditiously upon safety signals. In this case, we have not explored ways to preserve efficacy while mitigating risk, and this will be remembered as a failure of COVID-19 vaccine programs.

While it might have made sense to aggressively pursue vaccination in people at low risk of disease, if vaccination could create a state of hurt immunity, which would drive the virus into extinction, this hypothesis was not explicitly tested in randomized control trials of vaccination. And, many experts believed it was unlikely. Ergo, aggressively pursuing vaccination in young people, at even potential detriments to themselves, was an irrational policy goal.

## Natural Immunity

Early in the pandemic, two physicians wrote in the *New York Times* that antibodies were not destiny (Emanuel and Offit 2020). This turned out to be prescient for COVID-19, particularly among individuals who had experienced and recovered from the virus. Of course, for most adults, it is preferable to get the vaccine rather than the virus, but many individuals were unfortunately infected with sars-cov-2 prior to vaccination or boosting. For them, it is clear additional vaccine doses increase antibodies, but it is not clear whether vaccination—and how many shots—is needed to further lower risk of bad outcomes from COVID-19 reinfection.

Data suggest that having had and survived COVID-19 means the risk of bad outcomes following reinfection are staggeringly low. A paper that analyzes New York and California shows this clearly (León et al. 2022). Risks of hospitalization among those who had not had a previous COVID-19 diagnosis and were not vaccinated was 11.5 per 1000, while the risk of hospitalization for those who had a prior infection, regardless of vaccination, was 0.3 per 1000. The risk for those who were vaccinated without a prior diagnosis was 0.7 per 1,000. These results indicate that it is those who are unvaccinated without a prior infection, and not those who are unvaccinated with a prior infection who have a much higher relative risk of being hospitalized—somewhere between 2- and 17-times higher.

People who have been vaccinated or those who survived prior infection do not need proactive targeting by public health agencies. Instead, unvaccinated and uninfected adults deserve our focus. Yet, COVID-19 vaccine policies never acknowledged this. We could easily have accepted natural immunity as a vaccination equivalent, but the US CDC chose not to do this.

On an episode of a popular medical YouTube channel, the ZDoggMD show, vaccine researcher Paul Offit admitted that the White House conducted an informal poll to decide if natural immunity would count as a vaccine equivalent (ZdoggMD 2022), but this vote narrowly fell short. This error had severe negative repercussions for US faith in experts and public health and should not have been the subject of an informal, private vote.

## Mandates

An ethical prerequisite for mandating medical interventions is that there is sufficient benefit to others such that loss of individual autonomy is permissible. On Sept 9, 2021 (Fenyves 2022; Jamrozik et al. 2016), the Biden administration moved forward with vaccine mandates both for federal employees and private employees through OSHA regulation (Wingrove and Leonard 2021). Of course, as noted, polls showed that Republicans would be far more reluctant to embrace mandates than Democrats (Largent et al. 2020), and this action would further inject politics into vaccination campaigns.

More importantly, it is unclear whether the ethical prerequisite of benefit to others was met for vaccination. Available data in 2021 and early 2022 suggested that being vaccinated conferred tremendous personal benefit to the recipient, such that it was unclear if there could be added gain for demanding others be vaccinated too for added protection. By mid-2022, vaccines did offer modest reduction in transmission, but personal health benefits against severe disease were largely retained. Yet, by the fall of 2022, with the emergence of the Omicron variant, a new verdict had emerged. Vaccines were unable to halt transmission in the presence of escape variants; thus, here too, mandates failed to meet the ethical pre-requisite of benefit to others, as a vaccinated person could still spread the virus. A study in the New England Journal of Medicine showed comparable rates of viral shedding comparing vaccinated to unvaccinated people with COVID-19 (Boucau et al. 2022).

Even if one believed that mandates were ever ethically permissible, it is not clear they were wise public policy in a divided nation with strong political preferences around mandates (Prasad 2021b). First, gains in vaccination must be discounted by the secular trend, i.e., mandates should only be given credit for vaccination beyond what was expected in their absence. Second, gains from mandates would be offset by a fraction of employees being displaced from work—their negative socioeconomic and health outcomes must be added to the ledger. Third, mandates were an exercise of the sheer power of the federal government, and may still yield unanticipated effects, such as shifts in political power or voting preferences or erosion of trust in public health. These choices may erode health outcomes for decades to come.

One place where COVID-19 vaccine mandates have caused great consternation is as a prerequisite to attend public school. Famously, Los Angeles announced one such mandate. At the time, in US News and World Report, I argued that this would be unnecessarily draconian, disproportionately exclude minority and poor children from public education, and have unclear public health gain (Prasad 2021c). Ultimately, Los Angeles backed away from this proposal (Sequeira 2022), likely when it became evident the policy would disproportionately target black and Hispanic communities. A similarly misguided bill was offered at the California State level, but ultimately did not become law (Høeg and Prasad 2022). Yet, the fall of 2022, saw some districts—such as the District of Columbia—pursuing these policies, despite concern of disproportionate impact on black children (Holland and Johnson 2022).

Colleges took harsher actions. Many colleges rapidly embraced both vaccine and booster mandates (Bienen and Prasad 2022). Boosting requirements often did not contain exemptions for a prior or recent COVID-19 infection. It remains unclear and reasonably unlikely, that boosting a 20 year old healthy man who already had 2 doses and then gets breakthrough Omicron would benefit him or others, and yet that was precisely the requirement to attend in-person college (Makary 2021). Emboldened by federal mandates, schools and colleges pushed mandates of their own which seemed to defy logic. Thousands of students petitioned for these to be dropped (Brady 2022).

## Vaccine effectiveness against symptomatic disease (not severe disease) plummets

While vaccine effectiveness gradually fell during 2021—i.e., the ability of the vaccine to prevent any symptomatic disease was reduced—the rise of Omicron led to plummeting vaccine effectiveness, being reduced from about 50–90% to about 10% or lower (Cao et al. 2022). (Protection against severe disease remained strong.)

Of course, plummeting vaccine effectiveness has implications for the ethics of vaccine mandates (Prasad 2022a)—loss of autonomy is not justified if a vaccine cannot benefit a third party—as well as the use of vaccine passports (used by restaurants and bars or airline travel (in Canada) (Baral et al. 2021))—excluding individuals by vaccination status is not ethically permissible when vaccination cannot separate individuals who can spread from those who cannot.

Beyond this, the rise of immune evasive viral variants meant that no amount of vaccination would halt viral spread. The goals of vaccine campaigns, particularly additional doses, had to shift to focus solely on further reductions in severe disease—beyond what was achieved by the first 2 doses—and not merely to reduce symptomatic infection. Ironically, this was the original point by Topol and colleagues in 2020. This is true for a simple reason, that, if one lives long enough, with repeated exposure, infection and re-infection are inevitable, likely many times over a long life. Instead, avoiding bad outcomes is the only justification for repeated dosing of mRNA vaccination.

## Boosters and FDA resignations

In April 2021, the Pfizer CEO Albert Bourla announced that we would likely need boosters in the next 12 months (Lovelace 2021). In the months that followed, there was initially push back from the administration, including from Dr. Anthony Fauci. In July 2021 however, a private meeting would occur between senior administration officials and Pfizer, and from that moment forward, the White House began to push the message that a booster would be necessary.

Yet, two senior FDA officials were not persuaded—Marion Gruber and Philip Krause (aforementioned). These two served as director and deputy director of vaccine products for years and survived all 4 years under Trump. They authored a piece in the Lancet on Sept 13, 2021 critical of the evidence for universal boosters (Krause et al. 2021). They wrote, “Current evidence does not, therefore, appear to show a need for boosting in the general population, in which efficacy against severe disease remains high.”

Unfortunately, facing immense White House pressure to authorize a booster for all ages, the two resigned in protest (Collman 2021). Despite Trump’s many forward-looking statements on vaccine authorization, these two remained, but resigned in protest under his successor Joseph Biden. Of course, given plummeting vaccine effectiveness against symptomatic disease, it was possible that a 3rd dose would transiently lower the risk of breakthrough infection, but this would be beside the point. In a world where breakthrough is inevitable only severe disease, hospitalization, and death are suitable endpoints to judge the success of repeated vaccine injections. To date, there are no high-quality data supporting vaccines in young ages, which was the root of Gruber and Krause’s objection. This was further described in multiple op-eds by Dr. Krause (2021a, 2021b). Paul Offit famously advised his own son—in his twenties—not to receive the booster (Gutman‑Wei 2022).

## EUA in kids

COVID-19 was initially an emergency in adults, but for kids it was unclear. The UK’s Financial Times reported the infection fatality rate by age in two periods of time, 2020 and 2022 (Burn‑Murdoch and Barnes 2022). The analysis makes clear that in 2020, influenza had a comparable rate of death per infection than COVID-19, but by 2022, COVID-19 was far lower for children. An analysis from Germany found that the risk of death to a healthy child, during the height of the pandemic and pre-vaccine June 2020–May 2021, with COVID-19 was 3 in 1,000,000 (Sorg et al. 2021).

Whether or not kids faced an emergency has regulatory consequences. The use of the EUA pathway—which Dr. Hotez called “a substandard or lesser review” in 2020—hinges on whether an emergency is taking place. On May 7, 2021, with my colleagues Drs. Stefan Baral from Johns Hopkins and Wes Pegden from Carnegie Mellon, I wrote an essay in BMJ Opinion arguing that use of EUA pathways was not warranted for kids vaccine, and that the traditional biological licensing agreement pathway ought to be used (Pegden et al. 2021).

As mentioned, the argument for traditional approval had been advanced by the National Vaccine Advisory Committee for adult COVID-19 vaccination in Sept 2020, (Jenco 2020) but that argument made little sense at that moment, as COVID-19 posed tremendous risk to adults and was an emergency. Instead, for children the argument did hold. At the time we wrote, “Controversy surrounding mass child vaccination under emergency use authorizations could feed vaccine hesitancy in the United States at a time when public attitudes towards vaccination are critical.” This argument has proven prophetic as both the COVID-19 kids vaccine uptake has been low (~ 33% at the time of this writing), and we have seen a decline in routine childhood vaccination. The *New York Times* reports this is, in part, attributable to “a groundswell of resistance to COVID-19 shots spilling into unease about other vaccines.” (Mueller and Hoffman 2022).

Before the resignation of Gruber and Krause, in July 2021, the FDA had asked Pfizer and Moderna to expand the sample size of their kids vaccine studies (Stolberg et al. 2021). Kids vaccine trials used the primary endpoint of geometric mean antibody titers—i.e., antibody levels—and sought to show the antibody levels generated in kids were not inferior to those generated at older ages. The vaccine tested in kids was at a lower dose than in adults, but fundamentally directed against the original viral sequence isolated in Wuhan. The motivation for trial expansion was unlikely to further solidify knowledge of antibody levels, but more likely for exploration of safety and other, more relevant, measures of efficacy, such as rates of COVID-19 disease.

Arguably, the endpoint (antibody levels) of the kids’ vaccine trial was inadequate (Prasad 2021d). Parents and doctors did not merely want to know that the vaccine generated antibodies, but instead that vaccination lowered the risk of severe disease, death, or multi-inflammatory syndrome (MIS-c) in kids. As mentioned in the op-ed from June of 2020, antibodies alone were generally not sufficient to draw this conclusion. They especially were not sufficient against the backdrop of an evolving and changing virus. Prior commenters noted the much larger size of the rotavirus (60 k and 70 k +) and polio trials (400 k +), but the Pfizer trial in 5 to 11 year old children would begin with a plan to recruit just 2000 kids, and, after expansion, eventually enrolled under 5000 kids (MacMillan 2022). This sample size would be too low to draw any conclusions regarding whether, or to what degree, the vaccine protected against the endpoints that parents care about. Moreover, data from 2022 would show that, in the face of Omicron, the effectiveness of a kids vaccine would wane rapidly (Fleming‑Dutra et al. 2022). Again, the US FDA could have compelled Pfizer to conduct large randomized trials to definitively settle the question but did not.

In the absence of randomized studies, experts relied on a case–control design to infer that kids vaccines (in ages 5 to 11) lower severe disease (Price et al. 2022). Yet these studies contain a fatal flaw worth understanding. In a case control study, you select cases—kids hospitalized for COVID-19—and controls—kids hospitalized for something else—and ask how often each group received antecedent vaccination. If the controls have much higher rates than cases, the inference is that vaccination confers protection against disease.

Yet, this analysis hinges on the assumption that cases and controls are otherwise comparable. Are they? Controls—kids hospitalized for other reasons—also includes a subset of kids with severe underlying medical problems or those at risk for hospitalization. Some of these kids may be expected to be hospitalized beyond what we might expect for cases. Pediatricians and parents of these kids would be intensely interested in promoting vaccination in this group– because they know these kids are vulnerable—and thus a case control study that shows “vaccines lower severe disease” may in reality be showing little more than “parents of kids likely to be hospitalized were eager to vaccinate their kid.” It is difficult, if not impossible, to overcome this limitation in case–control studies, and other designs suffer from deep methodologic challenges beyond the scope of this essay. It is still uncertain whether, and to what degree, vaccinating kids 5 to 11 will reduce severe disease, hospitalization, MIS-c, and death.

## Months to 4-year-olds

**Fig. 2:**
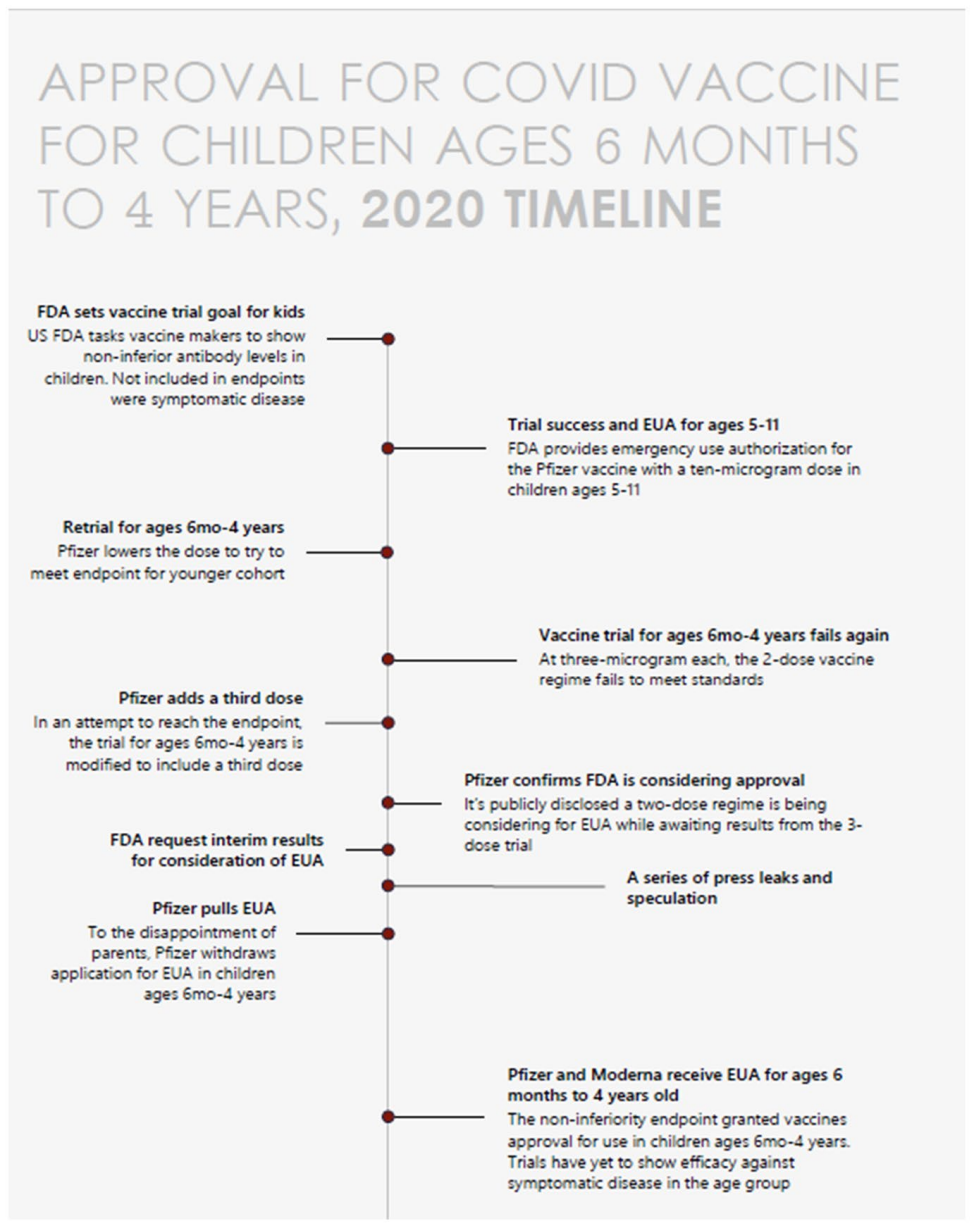
Timeline of approval for COVID vaccine for children, ages 6 months to 4 years (2020)

The growing list of COVID-19 vaccine errors—pushing it in populations at low risk, mandating it when ethical pre-requisites were not met, and failing to generate reliable, informative evidence—came to include vaccine approval for kids 6 months to 4 years old. The series of events around EUA in the United States raised countless concerns. I will highlight the most important ones, and refer readers to other essays that lay out the full case (Prasad 2022b). These are also shown in the timeline in Fig. 2.

First, the US FDA again tasked vaccine makers with showing non-inferior antibody levels, and not improvements in symptomatic disease, severe disease, hospitalization, MIS-c, or death. Next, Pfizer famously failed to meet even this modest bar. The company was permitted by FDA to add a third dose to the trial (Fox and Langmaid 2021). This protocol modification showed immense flexibility from regulators. If additional doses could be added until non-inferiority was reached, then there would be an increasing likelihood that the findings could occur by chance alone. Alternatively, the trial should have been halted and restarted with a higher dose.

In January 2022, a series of leaks suggested the FDA may have invited Pfizer to submit for EUA based on an interim analysis of symptomatic cases that favored the vaccine arm (Prasad 2022b). This would have been an astonishing and unprecedented lowering of regulatory standards (an unplanned look at an endpoint that was not the study’s primary endpoint), and the idea faced strong pushback. Ultimately on the cusp of an advisory meeting, Pfizer abruptly withdrew their EUA. This back and forth spanned a two-week period when some American parents got their hopes up, only to have them dashed. Vaccine regulators seemed unconcerned their erratic actions might spawn doubt in the American public.

Then, several months later in June of 2022, both Pfizer and Moderna ultimately received EUA for kids aged 6 months to 4 years old. Both companies reported meager vaccine efficacy against symptomatic disease in the face of Omicron. For Pfizer, the US FDA declined to endorse a specific numerical estimate of vaccine efficacy. The FDA writes, “An additional analysis pertaining to the occurrence of COVID-19 cases was determined not to be reliable due to the low number of COVID-19 cases that occurred in study participants” (US Food and Drug Administration 2022b). For Moderna, the agency endorsed a vaccine efficacy of 50.6% in kids 6 months to 2 years and 36.8% in kids aged 2 to 5 (US Food and Drug Administration 2022b).

However, one crucial development was, by the time these studies were conducted, that home based testing had gained popularity. The primary study analysis relied on laboratory testing and excluded home tests. If home tests were included (page 116), (US Food and Drug Administration, 2022a) the vaccine effectiveness for Moderna plummeted to 28.5%. Recall the argument from 2020, “A weakly effective vaccine can do more harm than good.”—that quote was from Phil Krause, one of the two resigned FDA reviewers, and that line of thinking and those standards had been entirely forgotten.

There is one more major issue with the vaccine trials in kids less than 5 years old. These studies included very few children who had previously recovered from COVID-19. That is in stark contrast to the reality in America according to the CDC’s own statistics (Edwards 2022), where at least 75% of kids already had COVID-19. As mentioned, natural immunity means that it is harder for someone to become reinfected with the virus and suffer a severe consequence. Frankly, the FDA has no reliable data that vaccinating a healthy child who already had COVID-19 with an old, ancestral Wuhan strain-based mRNA vaccine (the only vaccines used to date) lowers that child’s risk of severe disease, MIS-c, hospitalization, or death. As vaccines appear powerless, with time, to halt transmission, there is no evidence that vaccination benefits parents, grandparents, teachers, or the community. At the same time, myocarditis appears to be largely a post-pubescent phenomenon, and rates in children are lower than adolescents; thus, the vaccine is safer.

Ultimately, the FDA’s and CDC’s actions with kids’ vaccines are a complete medical, public health, and regulatory gamble with complex spill-over effects for other vaccines (Mueller and Hoffman 2022).

## A yearly covid shot?

We are rapidly moving toward a yearly vaccine taken in perpetuity—based on preclinical data, including animal studies, but without randomized trials measuring clinical outcomes. FDA leaders have sketched out this possibility in the Journal of the American Medical Association (Marks et al. 2022). And recently Reuters announced, “FDA will not require clinical trial data to authorize redesigned COVID boosters -official” (Erman 2022). The general idea is the agency will assign vaccine-makers a series of variant sequences they believe will be problematic in the fall and winter. Then, without trials proving that these vaccines lower the risk of bad outcomes, they will be debuted. Possibly, merely showing evidence of antibodies will be sufficient.

In the fall of 2022, this occurred with the development of the Bivalent Wuhan-Omicron BA.4/5 booster shot. This shot received emergency use authorization in the US, while the Bivalent Wuhan-Omicron BA.1 vaccine, which did have modest human data, received authorization in Canada. The US is notable for casting a wide net in booster authorizations—the vaccine product can be given to children as young as 12. The US campaign has not focused on those at highest risk—people over the age of 70 or those living in nursing care facilities.

In the fall of 2023, this was again repeated. While the UK (Department of Health and Social Care 2023), Sweden (The Public Health Agency of Sweden 2023), Denmark (Danske Regioner 2023), Spain (Soto 2023), Germany (Federal Ministry of Health 2023), Australia (Australian Government 2023) and other nations largely prioritized the vaccine in older adults (> 50 or > 65), the US embarked on a vaccination program for everyone over the age of 6 months (Zweig 2023). Notably, the former White House COVID-19 czar, Ashish Jha appeared on Good morning America advocating that a 20 year old man who had COVID 3 times and prior doses of vaccine, still should get this shot—a claim for which no evidence supports (Prasad 2023).

The decision to authorize a novel covid booster without human trials has 3 negative consequences. First, the precise mechanism of myocarditis has never been fully understood, and as such modifications may make the vaccine safer, or possibly more dangerous. There will be no mechanism to detect this before launch. Myocarditis seen with Novavax suggests that the spike itself is implicated and not the mRNA delivery mechanism. Second, there is no evidence we are better off from taking these vaccines. We don’t have trials measuring severe disease. Third, universities, employers, or the government may compel these yearly shots based on faulty reasoning, as a number of universities, including Tufts college (Mueller 2022), have already done.

Additionally, in an unusual action, FDA officials have advertised the shot with factually incorrect statements. The commissioner of the US FDA, Robert Califf tweeted, “The updated booster also increases your chances of being in attendance at upcoming gatherings with family and friends” (Califf @DrCaliff_FDA 2022). Of course, that statement is unsupported. Without human trials, he has no basis to make such a claim. Had the company said this, the FDA could fine them for false statements.

Paul Offit, a member of the FDA’s vaccine advisory committee, and a frequent commenter during the pandemic—disagreed with the FDA’s push for a yearly vaccine without clinical trials in all ages, and steadily found fault with the scientific basis in a series of op-eds and interviews (Fields 2022; Offit 2022).

The ultimate legacy of an untested, unproven, mandatory yearly shot will be to enrich the shareholders of pharmaceutical companies. Whether Americans will be better off, or which ones, remains unknown.

## Lessons Learned

The COVID-19 vaccine history carries many deep lessons. First it shows great ingenuity. Indeed, it is a scientific and technological accomplishment to develop COVID-19 vaccines in an unprecedented timespan, potentially saving tens of millions of lives globally (Watson et al. 2022). At the same time, vaccine development occurred during a contentious and divisive election year, and concern that the vaccine would be rushed to market prior to election day resulted in negative messaging in the media. Vaccine confidence in the American people declined during the election season. Petitions for enhanced safety follow up and greater events offered unclear scientific advantage and worked to delay authorization, raising questions regarding their motives. Perhaps some scientists and vaccine proponents were willing to play politics, even unknowingly, and their messaging shifted dramatically depending on who controlled the White House (Baral 2021).

A vaccine can be lifesaving and miraculous in high-risk populations, but that does not necessarily mean that giving more of it, giving it to younger populations, and giving it indefinitely is better. Carefully done randomized trials are needed to show whether we continue to benefit, or if we face diminishing returns—the flat of the curve (Mandrola et al. 2019). We need better evidence from large trials in populations that face the least risk from COVID-19, yet, ironically, we had the largest trials in adult populations. We tolerated less data for younger populations who have less to gain. Unanticipated safety signals are always possible, and 20,000 participants is not enough to exclude signals you do not anticipate (e.g., myocarditis). Regulators who are eager to deploy therapeutics should be equally quick to deal with unanticipated safety signals. To this day, we lack reliable information about the amount, number of doses, and timing of vaccination in young men that would minimize myocarditis. Finally, the role of mandates and their spillover effects remains controversial, as does a program that promotes a yearly COVID-19 vaccine for young healthy people.

Overall, these lessons should help inform future pandemic responses. Drug regulatory agencies can use the lesson learned from prior life-saving vaccines and drugs in setting efficacy and safety standards for those developed in times of emergency use. Protocols can be developed a priori that outline the implementation of randomized trials that can adaptively test questions of equipoise—namely the identification of population subgroups who are more or less likely to benefit, which can guide decisions about policy and treatment and prophylactics. Protocols for times of pandemic can also be developed a priori, which stipulate how to implement reporting and surveillance of disease-related information and adverse events. These data can be pulled to get nearly real-time information, which can help guide policy decisions and resource allocation, specifically in monitoring and responding to unknown safety signals. Finally, especially in the digital age where information is readily and publicly available, public health officials should be transparent in their evaluation of data related to public health policy.

COVID-19 vaccines: how can a single intervention that represents one of our greatest pandemic successes also capture many weaknesses of public policy? The answer is that vaccine development is a laboratory science exercise, under controlled and stable conditions, while policy occurs in the messy reality of the world—with complex human interactions, political forces, and diverse motivation. The COVID-19 vaccine has been both a story of the success of science and the failure of policy.

## References

[CR1] @VPrasadMDMPH (Vinay Prasad). 2021. *Yesterday I said the J&J vaccine is Game Over for women 18–50, with 6 cases in 1.4 million vaccinated in that age group (~1/200k), here is why I say that....* Twitter. https://twitter.com/vprasadmdmph/status/1382734864219140100.

[CR2] Andrews, N., E. Tessier, J. Stowe, C. Gower, F. Kirsebom, R. Simmons, E. Gallagher, S. Thelwall, N. Groves, G. Dabrera, R. Myers, C.N.J. Campbell, G. Amirthalingam, M. Edmunds, M. Zambon, K. Brown, S. Hopkins, M. Chand, S.N. Ladhani, and J. Lopez Bernal. 2022. Duration of protection against mild and severe disease by covid-19 vaccines. *The New England Journal of Medicine* 386 (4): 340–350. 10.1056/NEJMoa2115481. 35021002 10.1056/NEJMoa2115481 PMC8781262

[CR3] *Approval letter: BNT162b2*. 2021.

[CR4] Australian Government. 2023. *COVID-19 booster vaccine advice*. Australian Government Department of Health and Aged Care. https://www.health.gov.au/our-work/covid-19-vaccines/getting-your-vaccination/booster-doses

[CR5] Baden, L. R., H. M. El Sahly, B. Essink, K. Kotloff, S. Frey, R. Novak, D. Diemert, S. A. Spector, N. Rouphael, C. B. Creech, J. McGettigan, S. Khetan, N. Segall, J. Solis, A. Brosz, C. Fierro, H. Schwartz, K. Neuzil, L. Corey, COVE Study Group. 2021. Efficacy and safety of the mRNA-1273 SARS-CoV-2 vaccine. *The New England Journal of Medicine*, *384*(5), 403–416. 10.1056/NEJMoa2035389. 10.1056/NEJMoa2035389 PMC7787219 33378609

[CR6] Baral, S. (2021). *How did COVID-19 reach the White House?* Baltimore Sun. https://www.baltimoresun.com/opinion/op-ed/bs-ed-op-1008-politicizing-pandemic-20201007-yg6tsty6vjclvingwsjijqjhly-story.html.

[CR7] Baral, S. D., Rwema, J. O. T., and Phaswana-Mafuya, N. (2021). Covid-19 vaccine passports will harm sustainable development. *The BMJ*.

[CR8] Bienen, L., and V. Prasad. 2022. *Campus covid restrictions harm students*. City Journal. https://www.city-journal.org/campus-covid-restrictions-harm-students.

[CR9] Boucau, J., C. Marino, J. Regan, R. Uddin, M. C. Choudhary, J. P. Flynn, G. Chen, A. M. Stuckwisch, J. Mathews, M. Y. Liew, A. Singh, T. Lipiner, A. Kittilson, M. Melberg, Y. Li, R.F. Gilbert, Z. Reynolds, S. L. Iyer, G. C. Chamberlin, A. K. Barczak. 2022. Duration of shedding of culturable virus in SARS-CoV-2 omicron (BA.1) infection. *The New England Journal of Medicine*, *387*(3), 275–277. 10.1056/NEJMc2202092 10.1056/NEJMc2202092 PMC9258747 35767428

[CR10] Brady, E. 2022. *Thousands of College Students Are Petitioning Against Three Universities’ Booster Mandates*. Newsweek. https://www.newsweek.com/thousands-college-students-are-petitioning-against-three-universities-booster-mandates-1670818

[CR11] Branswell, H. 2022. *FDA limits use of Johnson & Johnson’s Covid-19 vaccine*. STAT. https://www.statnews.com/2022/05/05/fda-limits-use-of-johnson-johnsons-covid-19-vaccine/

[CR12] Buchan, S.A., C.Y. Seo, C. Johnson, S. Alley, J.C. Kwong, S. Nasreen, A. Calzavara, D. Lu, T.M. Harris, K. Yu, and S.E. Wilson. 2022. Epidemiology of myocarditis and pericarditis following mRNA vaccination by vaccine product, schedule, and interdose interval among adolescents and adults in Ontario, Canada. *JAMA Network Open* 5 (6): e2218505. 10.1001/jamanetworkopen.2022.18505. 35749115 10.1001/jamanetworkopen.2022.18505 PMC9233237

[CR13] Buchbinder, S.P., M.J. McElrath, C. Dieffenbach, and L. Corey. 2020. Use of adenovirus type-5 vectored vaccines: A cautionary tale. *The Lancet* 396 (10260): e68–e69. 10.1016/S0140-6736(20)32156-5. 10.1016/S0140-6736(20)32156-5 PMC7571904 33091364

[CR14] Burn-Murdoch, J., and O. Barnes. 2022. *Vaccines and Omicron mean Covid nnow less deadly than flu in England*. Financial Times. https://www.ft.com/content/e26c93a0-90e7-4dec-a796-3e25e94bc59b

[CR15] Califf @DrCaliff_FDA, R. M. 2022. *Being vaccinated and boosted reduces your risk of dying or getting critically ill and going to the hospital. The updated booster also increases your chances of being in attendance at upcoming gatherings with family and friends.* Twitter. https://twitter.com/DrCaliff_FDA/status/1568224957238284290?s=20&t=hJlYU6yrk38x47akkliDGw

[CR16] Cancryn, A., and S. Owermohle. 2020. *Pfizer trying to defuse critics amid push for vaccine before Election Day* . POLITICO. https://www.politico.com/news/2020/10/09/coronavirus-vaccine-pfizer-election-day-428371

[CR17] Cao, L., J. Lou, S.Y. Chan, H. Zheng, C. Liu, S. Zhao, Q. Li, C.K.P. Mok, R.W.Y. Chan, M.K.C. Chong, W.K.K. Wu, Z. Chen, E.L.Y. Wong, P.K.S. Chan, B.C.Y. Zee, E.K. Yeoh, and M.H. Wang. 2022. Rapid evaluation of COVID-19 vaccine effectiveness against symptomatic infection with SARS-CoV-2 variants by analysis of genetic distance. *Nature Medicine* 28 (8): 1715–1722. 10.1038/s41591-022-01877-1. 10.1038/s41591-022-01877-1 PMC9388371 35710987

[CR18] Cohen, J. 2020. Fact check: No evidence supports Trump’s claim that COVID-19 vaccine result was suppressed to sway election. *Science*. 10.1126/science.abf6577. 10.1126/science.abf6577

[CR19] Collman, A. 2021. *2 Top FDA officials resigned over biden’s booster plan, saying it insisted on the policy before the agency approved it, reports say*. Insider. https://www.businessinsider.com/2-top-fda-officials-resigned-biden-booster-plan-reports-2021-9

[CR20] Danske Regioner. 2023. *Everyone over the age of 65 can be vaccinated against influenza and covid-19 at the regions and pharmacies*. https://www.regioner.dk/services/nyheder/2023/september/alle-over-65-aar-kan-vaccineres-mod-influenza-og-covid-19-hos-regionerne-og-apotekerne/

[CR21] Department of Health and Social Care. 2023. *JCVI statement on the COVID-19 vaccination programme for autumn 2023—update 7 July 2023*. Gov.UK. https://www.gov.uk/government/publications/covid-19-autumn-2023-vaccination-programme-jcvi-update-7-july-2023/jcvi-statement-on-the-covid-19-vaccination-programme-for-autumn-2023-update-7-july-2023

[CR22] Doshi, P., and E. Topol. 2020. *Opinion | Coronavirus vaccine trials could suffer from shortcuts—The New York Times*. New York Times. https://www.nytimes.com/2020/09/22/opinion/covid-vaccine-coronavirus.html

[CR23] Edwards, E. 2022. *CDC report finds 75 percent of children and teens had Covid by February*. NBC News. https://www.nbcnews.com/health/health-news/cdc-says-75-percent-children-covid-february-rcna26029

[CR24] Emanuel, E., and P. Offit. 2020. Could trump turn a vaccine into a campaign stunt? *New York Times*.

[CR25] Erman, M. 2022. *FDA will not require clinical trial data to authorize redesigned COVID boosters -official* . Reuters. https://www.reuters.com/legal/government/fda-will-not-require-clinical-trial-data-authorize-redesigned-covid-boosters-2022-06-30/

[CR26] European Medicines Agency. 2021. *Meeting highlights from the pharmacovigilance risk assessment committee (PRAC) 3–6 May 2021*. European Medicines Agency. https://www.ema.europa.eu/en/news/meeting-highlights-pharmacovigilance-risk-assessment-committee-prac-3-6-may-2021

[CR27] Federal Ministry of Health. 2023. *Current information on coronavirus vaccination*. https://www.bundesgesundheitsministerium.de/en/coronavirus/faq-covid-19-vaccination.html

[CR28] Fenyves, P. 2022. *You have no mandate: A preemptive argument against mandating bivalent boosters*. Sensible Medicine. https://sensiblemed.substack.com/p/you-have-no-mandate-a-preemptive

[CR29] Fields, R. 2022. *The COVID-19 booster’s public relations problem*. Medscape. Retrieved October 4, 2022, from https://www.medscape.com/viewarticle/981491

[CR30] Fleming-Dutra, K.E., A. Britton, N. Shang, G. Derado, R. Link-Gelles, E.K. Accorsi, Z.R. Smith, J. Miller, J.R. Verani, and S.J. Schrag. 2022. Association of prior BNT162b2 COVID-19 vaccination with symptomatic SARS-CoV-2 infection in children and adolescents during omicron predominance. *The Journal of the American Medical Association* 327 (22): 2210–2219. 10.1001/jama.2022.7493. 35560036 10.1001/jama.2022.7493 PMC9107063

[CR31] Florko, N. 2022. *Ashish Jha to replace Zients as White House Covid response coordinator*. STAT. https://www.statnews.com/2022/03/17/ashish-jha-replace-jeff-zients-covid-coordinator/

[CR32] Fox, M., and V. Langmaid. 2021. *Pfizer’s child-sized vaccine fails to produce expected immunity in younger kids; company adds third dose to trials*. CNN. https://www.cnn.com/2021/12/17/health/pfizer-vaccine-children/index.html

[CR33] Goodnough, A., and J. Hoffman. 2020. *The elderly vs. essential workers: who should get the coronavirus vaccine first?*. The New York Times. https://www.nytimes.com/2020/12/05/health/covid-vaccine-first.html

[CR34] Government.no. 2022. *Vaccination of children and adolescents against COVID-19*. Government.No. https://www.regjeringen.no/en/aktuelt/vaccination-of-children-and-adolescents-against-covid-19/id2895513/

[CR35] Gray, E. 2021. *J&J Covid vaccine, birth control, and the inherent risk of modern medicine*. MSNBC. https://www.msnbc.com/opinion/j-j-covid-vaccine-birth-control-inherent-risk-modern-medicine-n1264013

[CR36] Greinacher, A., T. Thiele, T.E. Warkentin, K. Weisser, P.A. Kyrle, and S. Eichinger. 2021. Thrombotic thrombocytopenia after ChAdOx1 nCov-19 vaccination. *The New England Journal of Medicine* 384 (22): 2092–2101. 10.1056/NEJMoa2104840. 33835769 10.1056/NEJMoa2104840 PMC8095372

[CR37] Gumbrecht, J., and J. Christensen. 2022. *Covid-19 vaccine: Time between Pfizer and Moderna Covid-19 doses can be up to 8 weeks for some people, updated CDC guidance says*. CNN. https://www.cnn.com/2022/02/23/health/covid-vaccine-interval-cdc-guidance/index.html

[CR38] Gutman-Wei, R. 2022. *Should teens get a booster for omicron?* The Atlantic. https://www.theatlantic.com/health/archive/2022/01/should-teens-get-booster-omicron/621222/

[CR39] Haseltine, W. 2022. *Beware of covid-19 vaccine trials designed to succeed from the start*. Washington Post. https://www.washingtonpost.com/opinions/2020/09/22/beware-covid-19-vaccine-trials-designed-succeed-start/

[CR40] Herper, M. 2020a. *Experts see chance for Covid-19 vaccine approval this fall—if done right*. STAT. https://www.statnews.com/2020/09/02/experts-see-a-chance-for-a-covid-19-vaccine-approval-this-fall-if-its-done-right/

[CR41] Herper, M. 2020b. *Covid-19 vaccine from Pfizer and BioNTech is strongly effective, data show*. STAT. https://www.statnews.com/2020/11/09/covid-19-vaccine-from-pfizer-and-biontech-is-strongly-effective-early-data-from-large-trial-indicate/

[CR42] Høeg, T. B., V. Prasad, and M. Gandhi. 2021. *American kids can wait*. https://www.theatlantic.com/ideas/archive/2021/05/vaccinate-adults-in-india-before-american-children/618849/

[CR43] Høeg, T. B., and V. Prasad. 2022. *We’re pro-vaccine but can’t support California lawmaker’s school COVID vaccine mandate*. SFGate. https://www.sfgate.com/politics-op-eds/article/California-vaccine-mandate-schools-COVID-omicron-16832461.php

[CR44] Holland, E., and N. Johnson. 2022. *Covid vaccine mandates heighten school inequity*. The Wall Street Journal. Retrieved August 9, 2022, from https://www.wsj.com/articles/covid-vaccine-mandates-heighten-school-inequity-children-education-virus-pandemic-booster-public-health-students-11659992066?reflink=desktopwebshare_permalink&st=zlei2he3yqpsgje

[CR45] Hotez@PeterHotez, P. 2020. *New thread: A dozen reasons why I’m worried about releasing a #COVID19 #vaccine through an emergency use authorization (EUA)*. Twitter. https://twitter.com/peterhotez/status/1301275248248139778

[CR46] Jaffe-Hoffman, M. 2021. *19-year-old hospitalized in ICU days after getting second Pfizer vaccine* . The Jerusalem Post. https://www.jpost.com/health-science/19-year-old-hospitalized-with-heart-inflammation-after-pfizer-vaccination-657428

[CR47] Jamrozik, E., T. Handfield, and M.J. Selgelid. 2016. Victims, vectors and villains: Are those who opt out of vaccination morally responsible for the deaths of others? *Journal of Medical Ethics* 42 (12): 762–768. 10.1136/medethics-2015-103327. 27697791 10.1136/medethics-2015-103327 PMC5256398

[CR48] Jenco, M. (2020). Vaccine advisory group: Don’t rush COVID-19 vaccine approval. *AAP Publications*.

[CR49] Jha@ashishkjha, A. K. 2020. *@US_FDA chief @SteveFDA suggesting he could issue EUA for a vaccine if benefit > risk*. Twitter. https://twitter.com/ashishkjha/status/1300080022602969089?lang=ar

[CR50] Jiang, S. 2020. Don’t rush to deploy COVID-19 vaccines and drugs without sufficient safety guarantees. *Nature* 579 (7799): 321. 10.1038/d41586-020-00751-9. 32179860 10.1038/d41586-020-00751-9

[CR51] Johnson & Johnson. 2021. *Johnson & Johnson COVID-19 Vaccine Authorized by U.S. FDA For Emergency Use* . Johnson & Johnson. https://www.jnj.com/johnson-johnson-covid-19-vaccine-authorized-by-u-s-fda-for-emergency-usefirst-single-shot-vaccine-in-fight-against-global-pandemic

[CR52] Krause, P. 2021a. *We don’t need universal booster shots. We need to reach the unvaccinated.* The Washington Post. https://www.washingtonpost.com/outlook/2021/11/29/booster-shots-universal-opinion/

[CR53] Krause, P. 2021b. *The Biden administration has been sidelining vaccine experts*. The Washington Post. https://www.washingtonpost.com/outlook/2021/12/16/vaccines-fda-cdc-boosters-expert-panel/

[CR54] Krause, P.R., T.R. Fleming, R. Peto, I.M. Longini, J.P. Figueroa, J.A.C. Sterne, A. Cravioto, H. Rees, J.P.T. Higgins, I. Boutron, H. Pan, M.F. Gruber, N. Arora, F. Kazi, R. Gaspar, S. Swaminathan, M.J. Ryan, and A.-M. Henao-Restrepo. 2021. Considerations in boosting COVID-19 vaccine immune responses. *The Lancet* 398 (10308): 1377–1380. 10.1016/S0140-6736(21)02046-8. 10.1016/S0140-6736(21)02046-8 PMC8437678 34534516

[CR55] Krug, A., J. Stevenson, and T.B. Høeg. 2022. BNT162b2 vaccine-associated Myo/pericarditis in adolescents: A stratified risk-benefit analysis. *European Journal of Clinical Investigation* 52 (5): e13759. 10.1111/eci.13759. 35156705 10.1111/eci.13759 PMC9111575

[CR56] Largent, E.A., G. Persad, S. Sangenito, A. Glickman, C. Boyle, and E.J. Emanuel. 2020. US public attitudes toward COVID-19 vaccine mandates. *JAMA Network Open* 3 (12): e2033324. 10.1001/jamanetworkopen.2020.33324. 33337490 10.1001/jamanetworkopen.2020.33324 PMC7749443

[CR57] León, T.M., V. Dorabawila, L. Nelson, E. Lutterloh, U.E. Bauer, B. Backenson, M.T. Bassett, H. Henry, B. Bregman, C.M. Midgley, J.F. Myers, I.D. Plumb, H.E. Reese, R. Zhao, M. Briggs-Hagen, D. Hoefer, J.P. Watt, B.J. Silk, S. Jain, and E.S. Rosenberg. 2022. COVID-19 cases and hospitalizations by COVID-19 vaccination status and previous COVID-19 diagnosis—California and New York, May–November 2021. *MMWR. Morbidity and Mortality Weekly Report* 71 (4): 125–131. 10.15585/mmwr.mm7104e1. 35085222 10.15585/mmwr.mm7104e1 PMC9351527

[CR58] Letter to Pfizer. 2020.

[CR59] Lovelace, B. 2021. *Pfizer CEO says third Covid vaccine dose likely needed within 12 months*. CNBC. https://www.cnbc.com/2021/04/15/pfizer-ceo-says-third-covid-vaccine-dose-likely-needed-within-12-months.html

[CR60] Lovett, S. 2021. *Covid vaccine: UK decision to delay second dose vindicated by new research*. The Independent. https://www.independent.co.uk/news/science/covid-vaccine-second-dose-research-b1846417.html

[CR61] MacMillan, C. 2022. *COVID-19 vaccine authorized for kids ages 5 to 11: what parents need to know > News > Yale Medicine*. Yale Medicine. https://www.yalemedicine.org/news/covid-vaccine-for-ages-5-to-11

[CR62] Makary, M. 2021. *The dangerous push to give boosters to teens*. Wall Street Journal. https://www.wsj.com/articles/dangerous-push-to-give-boosters-to-teens-vacccine-covid-19-omicron-vaxx-requirement-mandate-11640107759

[CR63] Mandavilli, A. 2021. *CDC is investigating heart problems in a few young covid-19 vaccine recipients* . The New York Times. https://www.nytimes.com/2021/05/22/health/cdc-heart-teens-vaccination.html

[CR64] Mandrola, J., A. Cifu, V. Prasad, and A. Foy. 2019. The case for being a medical conservative. *The American Journal of Medicine* 132 (8): 900–901. 10.1016/j.amjmed.2019.02.005. 30851263 10.1016/j.amjmed.2019.02.005

[CR65] Marks, P., J. Woodcock, and R. Califf. 2022. COVID-19 vaccination-becoming part of the new normal. *The Journal of the American Medical Association* 327 (19): 1863–1864. 10.1001/jama.2022.7469. 35499853 10.1001/jama.2022.7469

[CR66] Martinez, D.R., and E.E. Ooi. 2022. A potential silver lining of delaying the second dose. *Nature Immunology* 23 (3): 349–351. 10.1038/s41590-022-01143-z. 35190721 10.1038/s41590-022-01143-z

[CR67] Mezher, M. 2020. *FDA issues EUA for Moderna COVID vaccine | RAPS*. Regulatory Focus. https://www.raps.org/news-and-articles/news-articles/2020/12/fda-issues-eua-for-moderna-covid-vaccine

[CR68] Miller, Z. 2021. *Biden urges shots for young adults as variant concern grows* . AP News. https://apnews.com/article/joe-biden-coronavirus-pandemic-health-government-and-politics-014b5b3d0bab3b6bc81aeabc898533e0

[CR69] Moderna’s COVID-19 Vaccine Candidate Meets its Primary Efficacy Endpoint in the First Interim Analysis of the Phase 3 COVE Study | Business Wire. 2020. Business Wire. https://www.businesswire.com/news/home/20201116005608/en/Moderna%E2%80%99s-COVID-19-Vaccine-Candidate-Meets-its-Primary-Efficacy-Endpoint-in-the-First-Interim-Analysis-of-the-Phase-3-COVE-Study

[CR70] Mueller, B., and J. Hoffman. 2022. *Routine childhood vaccinations in the U.S. slipped during the pandemic* . The New York Times. https://www.nytimes.com/2022/04/21/health/pandemic-childhood-vaccines.html

[CR71] Mueller, M. 2022. *As COVID-19 becomes endemic, Tufts transitions into new phase of public health response* . The Tufts Daily. https://tuftsdaily.com/news/2022/09/26/as-covid-19-becomes-endemic-tufts-transitions-into-new-phase-of-public-health-response/

[CR72] Nicholas, W., N. Sood, C.N. Lam, R. Kotha, H. Hu, and P. Simon. 2022. Did prioritizing essential workers help to achieve racial/ethnic equity in early COVID-19 vaccine distribution? The LA pandemic surveillance cohort study. *American Journal of Industrial Medicine* 65 (4): 231–241. 10.1002/ajim.23335. 35187706 10.1002/ajim.23335 PMC9082038

[CR73] Norwegian Institute of Public Health. (2022). *Coronavirus vaccine forchildren 5–11 years*.

[CR74] NPR. 2020. *Trump contradicts CDC director On COVID-19 vaccine distribution : coronavirus updates*. NPR. https://www.npr.org/sections/coronavirus-live-updates/2020/09/16/913560563/cdc-director-says-covid-vaccine-likely-wont-be-widely-available-until-next-year

[CR75] Offit, P. A. 2022. *CDC oversells the ‘Bivalent’ Covid Shot*. Wall Street Journal. https://www.wsj.com/articles/cdc-oversells-the-bivalent-covid-shot-hospitalizations-vaccine-booster-omicron-pandemic-pfizer-moderna-china-illness-death-11663793472

[CR76] Pegden, W., V. Prasad, and S. Baral, S. (2021). Covid vaccines for children should not get emergency use authorization. *The BMJ*.

[CR77] Pfizer. 2020a. *Pfizer and BioNTech announce vaccine candidate against COVID-19 achieved success in first interim analysis from phase 3 study* . Pfizer. https://www.pfizer.com/news/press-release/press-release-detail/pfizer-and-biontech-announce-vaccine-candidate-against

[CR78] Pfizer. 2020b. *Pfizer and BioNTech celebrate historic first authorization in the U.S. of vaccine to prevent COVID-19* . Pfizer. https://www.pfizer.com/news/press-release/press-release-detail/pfizer-and-biontech-celebrate-historic-first-authorization

[CR79] Prasad, V., R. Farzaneh-Far, W. Pegden, V. Murthy, and A. Beck. 2021. *CDC’s all-or-nothing approach to teen COVID vaccination is all wrong* . MedPage Today. https://www.medpagetoday.com/opinion/second-opinions/93340

[CR80] Prasad, V. 2021a. *COVID vax opponents and rigid proponents...are both anti-science?*. MedPage Today. https://www.medpagetoday.com/infectiousdisease/covid19vaccine/92413

[CR81] Prasad, V. 2021b. *Vax passports are a bad idea*. MedPage Today. https://www.medpagetoday.com/opinion/vinay-prasad/92107

[CR82] Prasad, V. 2021c. *Opinion: COVID-19 vaccines are good, but shouldn’t be mandated for school kids*. US News and World Report. https://www.usnews.com/opinion/articles/2021-09-28/opinion-covid-19-vaccines-are-good-but-shouldnt-be-mandated-for-school-kids

[CR83] Prasad, V. 2021d. *Doctors must be honest with parents about unknown risks of COVID-19 emergency vaccine*. Yahoo!News. https://news.yahoo.com/doctors-must-honest-parents-unknown-100213768.html

[CR84] Prasad, V. 2022a. *Vaccine effectiveness (against infection not severe disease) goes down the drain*. Vinay Prasad’s Observations and Thoughts. https://vinayprasadmdmph.substack.com/p/vaccine-effectiveness-goes-down-the

[CR85] Prasad, V. 2022b. *Another FDA blunder: How the food and drug administration botched the vaccine-approval process for young children*. City Journal. https://www.city-journal.org/how-the-fda-botched-the-vaccine-rollout-for-young-kids

[CR86] Prasad, V. 2023. *The Fall COVID19 shot is already a US Public Health Disaster*. Vinay Prasad’s Observations and Thoughts | Substack. https://vinayprasadmdmph.substack.com/

[CR87] Price, A. M., S. M. Olson, M. M. Newhams, N. B. Halasa, J. A. Boom, L. C. Sahni, P. S. Pannaraj, K. Irby, K. E. Bline, A. B. Maddux, R. A. Nofziger, M. A. Cameron, T. C. Walker, S. P. Schwartz, E. H. Mack, L. Smallcomb, J. E. Schuster, C. V. Hobbs, S. Kamidani, and Overcoming Covid-19 Investigators. 2022. BNT162b2 protection against the omicron variant in children and adolescents. *The New England Journal of Medicine*, *386*(20), 1899–1909. 10.1056/NEJMoa2202826 10.1056/NEJMoa2202826 PMC9006785 35353976

[CR88] Rasmussen@angie_rasmussen, A. 2021. *For perspective, here are some numbers:1 in 1,000,000: J&J vaccine1 in 3000: oral contraceptives1 in 5: hospitalized COVID-19 patients*. Twitter. https://twitter.com/angie_rasmussen/status/1381967413944623109

[CR89] Regalado, A. 2020. *One doctor’s campaign to stop a covid-19 vaccine being rushed through before Election Day* . MIT Technology Review. https://www.technologyreview.com/2020/10/19/1010646/campaign-stop-covid-19-vaccine-trump-election-day/

[CR90] Reich, J., and S. Masket. 2020. Trump’s rush for a covid vaccine could make it less likely to work . *The Washington Post*.

[CR91] Reuters. 2021a. *Israel examining heart inflammation cases in people who received Pfizer COVID shot*. Reuters. https://www.reuters.com/world/middle-east/israel-examining-heart-inflammation-cases-people-who-received-pfizer-covid-shot-2021-04-25/

[CR92] Reuters. 2021b. *U.S. CDC has not seen link between heart inflammation and COVID-19 vaccines* . Reuters. https://www.reuters.com/business/healthcare-pharmaceuticals/us-cdc-has-not-seen-link-between-heart-inflammation-covid-19-vaccines-2021-04-27/

[CR93] Reuters. 2021c. *Norway speeds up COVID-19 vaccination amid ample supply* . Reuters. https://www.reuters.com/business/healthcare-pharmaceuticals/norway-shortens-interval-between-covid-19-vaccine-doses-2021-06-07/

[CR94] Reuters. 2021d. *Finland joins Sweden and Denmark in limiting Moderna COVID-19 vaccine*. Reuters. https://www.reuters.com/world/europe/finland-pauses-use-moderna-covid-19-vaccine-young-men-2021-10-07/

[CR95] Romero-Brufau, S., A. Chopra, A.J. Ryu, E. Gel, R. Raskar, W. Kremers, K.S. Anderson, J. Subramanian, B. Krishnamurthy, A. Singh, K. Pasupathy, Y. Dong, J.C. O’Horo, W.R. Wilson, O. Mitchell, and T.C. Kingsley. 2021. Public health impact of delaying second dose of BNT162b2 or mRNA-1273 covid-19 vaccine: Simulation agent based modeling study. *BMJ (clinical Research Ed.)* 373: n1087. 10.1136/bmj.n1087. 10.1136/bmj.n1087 PMC8114182 33980718

[CR96] Sadoff, J., G. Gray, A. Vandebosch, V. Cárdenas, G. Shukarev, B. Grinsztejn, P. A. Goepfert, C. Truyers, H. Fennema, B. Spiessens, K. Offergeld, G. Scheper, K. L. Taylor, M. L. Robb, J. Treanor, D. H. Barouch, J. Stoddard, M. F. Ryser, M. A. Marovich, and ENSEMBLE Study Group. 2021. Safety and efficacy of single-dose Ad26.COV2.S vaccine against covid-19. *The New England Journal of Medicine*, *384*(23), 2187–2201. 10.1056/NEJMoa2101544 10.1056/NEJMoa2101544 PMC8220996 33882225

[CR97] Sepkowitz, K. 2020. *Why trump should worry about a rushed Covid vaccine (opinion)*. CNN. https://www.cnn.com/2020/09/10/opinions/trumps-rush-to-a-covid-vaccine-risks-this-danger-sepkowitz/index.html

[CR98] Sequeira, K. 2022. *Los Angeles unified recommends delaying vaccine mandate* . EdSource. https://edsource.org/updates/los-angeles-unified-recommends-delaying-vaccine-mandate

[CR99] Sharff, K.A., D.M. Dancoes, J.L. Longueil, P.F. Lewis, and E.S. Johnson. 2022. Surveillance of myopericarditis following COVID-19 booster dose vaccination in a large integrated health system. *MedRxiv*. 10.1101/2022.02.08.22270635. 10.1101/2022.02.08.22270635

[CR100] Sorg, A.L., M. Hufnagel, M. Doenhardt, N. Diffloth, H. Schroten, R.V. Kries, R. Berner, and J. Armann. 2021. Risk of hospitalization, severe disease, and mortality due to COVID-19 and PIMS-TS in children with SARS-CoV-2 infection in Germany. *MedRxiv*. 10.1101/2021.11.30.21267048. 10.1101/2021.11.30.21267048

[CR101] Soto, Á. 2023. *Fifth Covid jab for over 60s and vulnerable recommended by Ministry of Health in Spain* . Sur in English. https://www.surinenglish.com/spain/fifth-covid-jab-for-over-60s-and-20230828110715-nt.html

[CR102] Stolberg, S. G., LaFraniere, S., and Weiland, N. 2021. *Pfizer and moderna are expanding vaccine studies of kids 5 to 11* . The New York Times. https://www.nytimes.com/2021/07/26/us/politics/fda-covid-vaccine-trials-children.html

[CR103] Stolberg, S. G., and S. LaFraniere. 2021. *Warning of shortages, researchers look to stretch vaccine supply*. The New York Times. https://www.nytimes.com/2021/01/05/us/politics/coronavirus-vaccine-supply.html

[CR104] Taylor, C. 2021. *Nordic countries are restricting the use of Moderna’s Covid vaccine*. CNBC. https://www.cnbc.com/2021/10/08/nordic-countries-are-restricting-the-use-of-modernas-covid-vaccine.html

[CR105] The Public Health Agency of Sweden. 2023. *COVID-19*. https://www.folkhalsomyndigheten.se/the-public-health-agency-of-sweden/

[CR106] Topol, E. J., and P. A. Offit. 2020. *Paul Offit’s biggest concern about COVID vaccines*. Medscape. https://www.medscape.com/viewarticle/936937

[CR107] Torreele, E. 2020. The rush to create a covid-19 vaccine may do more harm than good . *The BMJ*. 10.1136/bmj.m3209 32816760

[CR108] Tuite, A.R., L. Zhu, D.N. Fisman, and J.A. Salomon. 2021. Alternative dose allocation strategies to increase benefits from constrained COVID-19 vaccine supply. *Annals of Internal Medicine* 174 (4): 570–572. 10.7326/M20-8137. 33395334 10.7326/M20-8137 PMC7808325

[CR109] Tyson, A., C. Johson and C. Funk. 2020. *U.S. public now divided over whether to get COVID-19 vaccine*. Pew Research Center. https://www.pewresearch.org/science/2020/09/17/u-s-public-now-divided-over-whether-to-get-covid-19-vaccine/

[CR110] US Food and Drug Administration. 2022a. *VRBPAC briefing document: moderna COVID-19 vaccine EUA amendment for use in children 6 months through17 years of age*.

[CR111] US Food and Drug Administration. 2022b. *Coronavirus (COVID-19) Update: FDA Authorizes Moderna and Pfizer-BioNTech COVID-19 Vaccines for Children Down to 6 Months of Age* . US Food and Drug. https://www.fda.gov/news-events/press-announcements/coronavirus-covid-19-update-fda-authorizes-moderna-and-pfizer-biontech-covid-19-vaccines-children

[CR112] Wachter, R. M., and A. Jha. 2021. *Its time to consider delaying the second dose coronavirus vaccine*. The Washington Post. https://www.washingtonpost.com/opinions/2021/01/03/its-time-consider-delaying-second-dose-coronavirus-vaccine/

[CR113] Warren, H., and D. Pogkas. 2020. *Covid vaccine: How the U.K. Will Distribute Pfizer, BioNTech Doses*. Bloomberg. https://www.bloomberg.com/graphics/2020-uk-vaccine-logistics/

[CR114] Watson, O.J., G. Barnsley, J. Toor, A.B. Hogan, P. Winskill, and A.C. Ghani. 2022. Global impact of the first year of COVID-19 vaccination: A mathematical modelling study. *The Lancet Infectious Diseases* 22 (9): 1293–1302. 10.1016/S1473-3099(22)00320-6. 35753318 10.1016/S1473-3099(22)00320-6 PMC9225255

[CR115] Wingrove, J., and J. Leonard. 2021. *Biden speech: Vaccines mandated for federal workers, contractors, millions more*. Bloomberg. https://www.bloomberg.com/news/articles/2021-09-09/biden-to-sign-order-requiring-vaccines-for-federal-workers

[CR116] World Health Organization. 2021. *Interim statement on COVID-19 vaccination for children and adolescents*. World Health Organization. https://www.who.int/news/item/24-11-2021-interim-statement-on-covid-19-vaccination-for-children-and-adolescents

[CR117] ZDoggMD. 2022. *Vaccine nonsense, debunked (w/Dr. Paul Offit)*. YouTube. https://www.youtube.com/watch?v=wkz1ln5AJ5Q

[CR118] Zimmer, C., and N. Weiland. 2020. *In reversal, white house approves stricter guidelines for vaccine makers—The New York Times*. New York Times. https://www.nytimes.com/2020/10/06/health/covid-vaccine-guidelines.html?searchResultPosition=65

[CR119] Zimmer, C. 2020. *The first covid vaccine will not make life normal again* . The New York Times. https://www.nytimes.com/2020/10/12/health/covid-vaccines.html?searchResultPosition=123

[CR120] Zweig, D. 2023. *The nyt publishes falsehood by former biden covid coordinator about UK vaccine policy*. https://www.silentlunch.net/p/the-nyt-publishes-falsehood-by-former

